# Primary Retention of Molars and RANKL Signaling Alteration during Craniofacial Growth

**DOI:** 10.3390/jcm9040898

**Published:** 2020-03-25

**Authors:** Andrea Gama, Laura Maman, Jorge William Vargas-Franco, Rana Omar, Bénédicte Brounais-Le Royer, Hideo Yagita, Sylvie Babajko, Ariane Berdal, Ana Carolina Acevedo, Dominique Heymann, Frédéric Lézot, Beatriz Castaneda

**Affiliations:** 1 INSERM, UMR-1138, Centre de Recherche des Cordeliers, Université de Paris, Sorbonne Université, Physiopathologie Orale Moléculaire, F-75006 Paris, France; dea.gama10@gmail.com (A.G.); ran.omar@gmail.com (R.O.); sylvie.babajko@inserm.fr (S.B.); ariane.berdal@univ-paris-diderot.fr (A.B.); 2 Odontology Center of District Federal Military Police, Brasília 70297-400, Brazil; 3 Oral Histopathology Laboratory, Health Sciences Faculty, University of Brasília, Brasília 70910-900, Brazil; 4 Service d’Odontologie-Stomatologie, Hôpital Pitié-Salpêtrière, AP-HP, F-75013 Paris, France; lau.maman@hotmail.com; 5 INSERM, UMR-1238, Equipe 1, Faculté de Médecine, Université de Nantes, F-44035 Nantes, France; jorge.vargas@udea.edu.co (J.W.V.-F.);; 6 Department of Basic Studies, Faculty of Odontology, University of Antioquia, Medellin A.A1226, Colombia; 7 Department of Immunology, Juntendo University School of Medicine, Tokyo 113-8421, Japan; hyagita@med.juntendo.ac.jp; 8 INSERM, LEA Sarcoma Research Unit, University of Sheffield, Department of Oncology and Human Metabolism, Medical School, Sheffield S10 2RX, UK; dominique.heymann@univ-nantes.fr; 9 INSERM, UMR 1232, LabCT, CRCNA, Université de Nantes, Université d’Angers, Institut de Cancérologie de l’Ouest, site René Gauducheau, F-44805 Saint-Herblain, France

**Keywords:** primary retention of molars, RANKL, craniofacial, growth

## Abstract

The primary retention of molars observed in clinic corresponds to a still-unexplained absence of molar eruption despite the presence of an eruption pathway, resembling the experimental transient inhibition of RANKL signaling in mice. The aim of the present study was to confront the hypothesis according to which the primary retention of molars is associated with transitory perturbations to RANKL signaling during growth as part of a wider craniofacial skeleton pattern. The experimental strategy was based on combining a clinical study and an animal study corresponding to the characterization of the craniofacial phenotypes of patients with primary retention of molars and analyses in mice of the consequences of transient inhibition of RANKL signaling on molar eruption and craniofacial growth. The clinical study validated the existence of a particular craniofacial phenotype in patients with primary retention of molars: a retromandibular skeletal class II typology with reduced mandibular dimensions which manifests itself at the dental level by a class II/2 with palatoversion of the upper incisors and anterior overbite. The animal study demonstrated that transient invalidation of RANKL signaling had an impact on the molar eruption process, the severity of which was dependent on the period of inhibition and was associated with a reduction in two craniofacial morphometric parameters: total skull length and craniofacial vault length. In conclusion, primary retention of molars may be proposed as part of the craniofacial skeleton phenotype associated with a transitory alteration in RANKL signaling during growth.

## 1. Introduction

Dental eruption is defined as the axial or occlusal movement of a tooth from its developmental position within the jaw towards its functional position within the occlusal plane [1]. Eruption disturbances may have systemic or local origins. Systemic origins are observed in patients with certain developmental syndromes [2,3] and usually, multiple teeth are affected. In contrast, when few permanent teeth are affected, and predominantly third molars and permanent canines, this is characteristic of local origins. Eruption disturbances to permanent first and second molars are relatively rare [3,4] but have a considerable clinical impact. Their normal eruptions are particularly important for craniofacial growth as a whole [5].

Primary and secondary retentions and impaction are the terms classically used to identify the different eruption disorders to permanent molars [4]. Impaction is a term reserved for teeth that are unable to erupt due to some physical barrier located on the eruption pathway [2]. A term often used in the literature to describe this event is Mechanical Failure of Eruption (MFE). In this disorder, there is no formation of an eruption pathway and teeth neighboring the impacted tooth are not affected. Common factors in the etiology of impacted teeth include lack of space due to crowding of the dental arches and premature loss of deciduous teeth. Secondary retention refers to the cessation of eruption after emergence. The molars remain in infra-occlusion at an age at which the tooth would normally be in occlusion. The etiopathogenesis of secondary retention is unknown but the involvement of ankylosis has been suggested [2,4]. The term primary retention is used to describe the incomplete eruption of a tooth that is normally positioned and that has developed in the absence of any obstacle on the eruption pathway [6]. Primary Failure of Eruption (PFE) is primary retention characterized by a posterior unilateral/bilateral open bite [7]. In this pathology, posterior teeth are commonly the most affected, and typically, all teeth distal to the main affected teeth exhibit the disorder. Part of the PFE corresponds to familial/genetic forms with mutations in the PTH1R gene identified [8], but the underlying mechanism is still unknown. Regarding the non-genetic forms, their origins remain elusive.

The Receptor Activator of Nuclear Factor kB Ligand (RANKL, also called TNFSF11), and its receptors RANK (also named TNFRSF11A) and OPG (osteoprotegerin or TNFRSF11B), are the main signals controlling osteoclastogenesis. RANKL, which is mainly synthesized by stromal cells, is an activator of osteoclastic differentiation through its binding to RANK at the surface of osteoclast precursor cells [9]. OPG is a soluble protein that can bind to RANKL with high affinity and compete with RANK, so it acts as an inhibitor of the RANKL effect on osteoclastogenesis [10]. Studies have demonstrated the direct involvement of RANKL/RANK/OPG signaling in the morphogenesis of the craniofacial skeleton and its growth, including control of the alveolar bone remodeling necessary for tooth growth and eruption [11,12,13]. Interestingly, in mice, RANK overexpression in the monocyte/macrophage lineage (*Rank^Tg^*) induces increased osteoclastic activity and premature dental eruption, while partial RANKL invalidation (*Rankl^+/-^*) induces decreased osteoclastic activity and a delay in dental eruption [14]. Moreover, several preclinical studies in mice [15,16] have evidenced that transitory inhibition of bone resorption during growth, obtained either with a bisphosphonate (zoledronic acid) or with an efficient RANKL blocking antibody (IK22.5), induced several craniofacial skeleton defects, including a blockage of tooth eruption. This blockage was further observed as a side effect in some of the young patients treated with bisphosphonates in an onco-pediatric context or in cases of osteogenesis imperfecta [17]. Additional preclinical studies in mice, consisting of grade shifting in the initiation of transitory inhibition of RANKL signaling, obtained with the IK22.5 antibody, evidenced that depending on age at the first injection, primary retention concerning only the first or the second molars could be obtained [13]. This result opens up new perspectives in the understanding of the origins of molar retention. Any transitory alterations in RANKL signaling during growth may be at the origin of molar eruption disturbances in the context of a wider spectrum of craniofacial skeleton defects. 

Consequently, regarding the origins of the Primary Retentions of Molars (PRM), the RANKL signaling status may be proposed as a central promoting element [13]. The main hypothesis of the present studies was that during childhood, alterations to bone resorption associated with transitory RANKL signaling perturbations, regardless of their origin (genetic or environmental), were the condition for the PRM. In order to test this hypothesis, the craniofacial skeleton phenotypes of patients with PRM were analyzed using both Tweed’s and Delaire’s morphometric analyses to characterize the specific features associated with PRM. In parallel, the craniofacial skeleton phenotypes of mice invalidated globally or transitorily for RANKL were analyzed and the results compared with those obtained for PRM patients in order to link PRM to singular craniofacial growth defects, all being secondary to transitory perturbations to RANKL signaling.

## 2. Experimental Section

### 2.1. Clinical Study

In order associate primary retention of molars (PRM) with a broader spectrum of craniofacial growth alterations, the craniofacial phenotypes of patients presenting with PRM and those with impactions or mechanical retentions were analyzed and compared. To do so, the orthodontic records of patients at the Dentofacial Orthopedics Department at the Hospital Pitié-Salpêtrière (AP-HP, Paris, France) were assessed in accordance with a declaration made to the French “Commission National de l’Informatique et des Libertés” (French data protection authority, number g8w2314849D).

For inclusion of the patients with retention, the criteria were (i) interrupted eruption of at least one permanent first or second molar (third and deciduous molars were excluded) with or without the presence of a physical obstacle on the eruption pathway, and (ii) patients at the young adult teeth stage at least. For the exclusion criteria of the patients, the criteria were (i) patients with medical syndromes involving eruption defects, such as cleidocranial dysplasia or bone diseases such as osteopetrosis, (ii) patients with a past history of orthodontic treatment and (iii) patients with an incomplete orthodontic file (missing x-rays).

Cohort studied: a total of 42 patients with eruption alterations to the first and second molars were included in the study. The patients were divided into two groups:

- The PRM group was composed of 24 patients with primary retention of molars. No mechanical obstacle to the eruption was visible, either clinically or radiographically, and the molars were positioned normally. The patients in this group were, on average, 16.75 years old, with an age range of 12 to 22 years (Table 1).

- The control (C) group was composed of 18 patients with mechanical impactions, representing the referent group. A physical obstacle or an axis unfavorable to the eruption of the molar could be objectified at the clinical or radiological levels. The average age of these patients was 16.25 years with an age range of 13.5 to 24 years (Table 1).

The patients’ distribution in terms of age, sex, ethnicity, family history (alteration to molar eruptions), uni- or bilateral characteristics, type of arcade affected, type of tooth affected, supra- or infra-crestal position, presence of a pathway of radiological eruption (for infra-crestal molars), other associated anomalies and the dental diagnosis are shown in Table 1.

**Figure 1:**
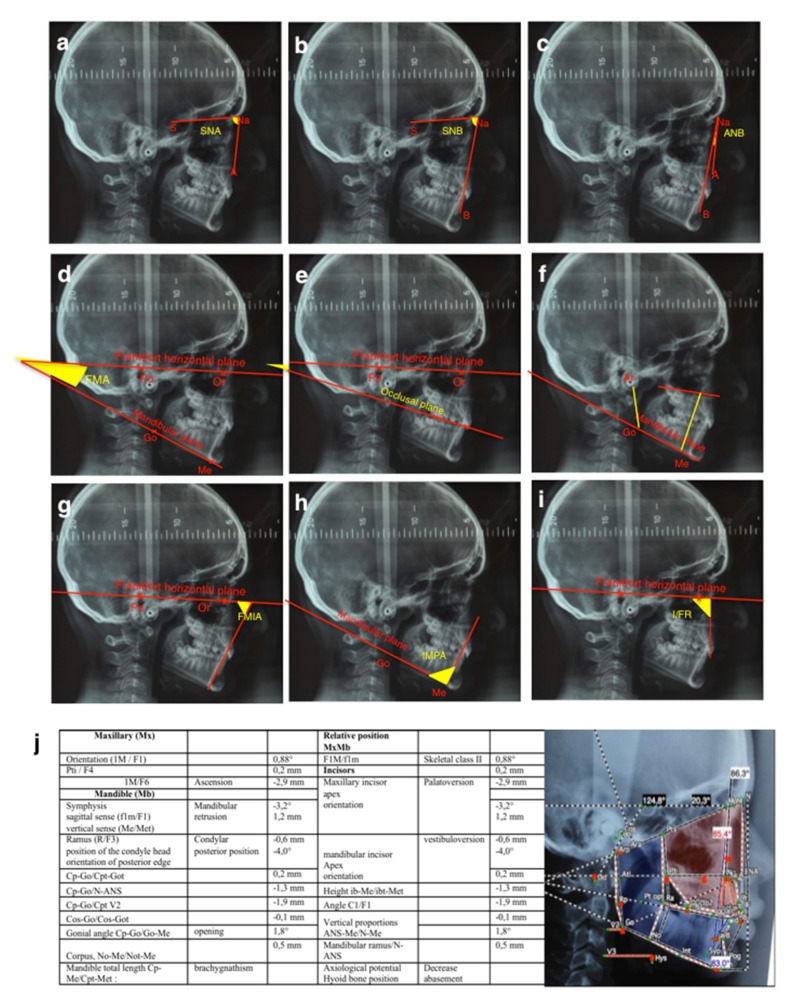
Cephalometric landmarks of Tweed’s (a-i) and Delaire’s (j) analyses. For Tweed’s analysis, the antero-posterior skeletal measurements were (**a**) the SNA, corresponding to the antero-posterior position of the maxilla in relation to the anterior cranial base, (**b**) the SNB, corresponding to the antero-posterior position of the mandible in relation to the anterior cranial base, and (**c**) the ANB, corresponding to the discrepancy between the maxilla and the mandible. For the vertical skeletal measurements, the FMA (**d**) corresponds to the Frankfort mandibular plane angle. (**e**) and (**f**) present respectively the occlusal plane/Frankfort angle and the vertical index measurements. (Ar: Articular point; Go: Gonion; Me: Menton). Concerning the dental measurements, (**g**) FMIA corresponds to the Frankfort mandibular incisor angle, (**h**) IMPA to the incisor mandibular plane angle and (**i**) I/FR to the upper incisor/Frankfort angle. For Delaire’s cephalometric analysis, (**j**) the table summarizes the different measurements made, as presented on the annotated tele-X-rays based on the positions of the following referent points: M, Metanasion; F1, anterior craniofacial balance; F4, craniopalatal point; C1, craniofacial base line; F, frontal; Op, posterior occipital point; Clp, posterior clinoid process; CT, temporal condylar point; Pts, superior pterygoid point; Na, nasion projected; ANS, anterior nasal spine; Go, gonion; FM, frontomaxillary articulation; Cp, posterior tangent of the condyle; Pti, inferior pterygoid point; NPC, naso-palatine canal; Me, menton; Bo, Bolton point; Od, odontoid; Om, mandibular-occipital point.

The tele-X-rays of these patients were studied using two different cephalometric analyses, the Tweed quantitative analysis (Charles Tweed International Foundation– CEPH, Orqual) (Figure 1a–g) and the Delaire qualitative analysis [18,19] (Software Delaire 2015 Evolution® v1.0, JDel, Nantes, France) (Figure 1h).

### 2.2. Mouse Models

All mice were housed in pathogen-free conditions at the Experimental Therapy Unit at the Faculty of Medicine at the University of Nantes (Nantes, France) in accordance with the institutional European guidelines (EU directive 2010/63/EU). All protocols applied in the present study were first validated by the French ethical committee of the “Pays de la Loire” (CEEA-PdL-06) and authorized by the French ministry of agriculture and fisheries (authorization numbers 11208-2017083115577055 and 18415-201901101823350-v2), under the supervision of authorized investigators.

For perinatal injections of RANKL blocking antibody (IK22.5) protocols, pregnant C57BL/6 mice (14 days of gestation) were purchased from Janvier’s Laboratories (Le Genest Saint Isle, France).

The *Rankl^-/-^* mice used in the present study were initially generated by Y. Choi [20]. Genotyping was ensured by PCR using the following primers *5’Rankl*: CCA AGT AGT GGA TTC TAA ATC CTG, *3’Rankl*: CCA ACC TGT GGA CTT ACG ATT AAA G and *3’insert*: ATT CGC AGC GCA TCG CCT TCT ATC as previously described [21].

**Figure 2:**
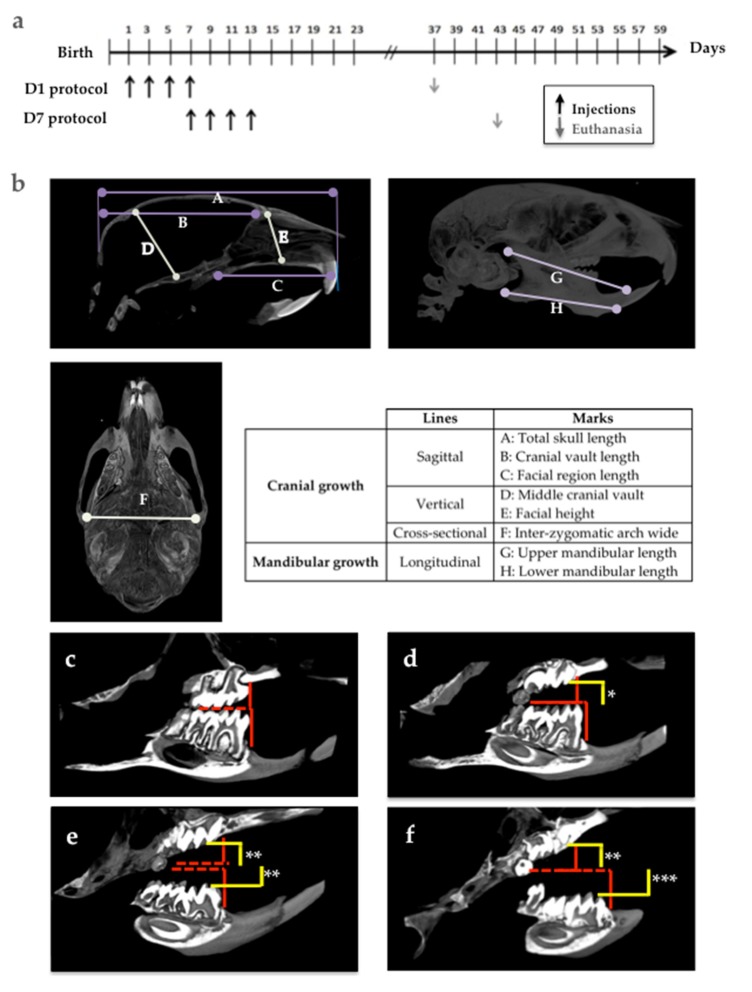
Experimental protocols for the IK22.5 antibody injections, definition of the morphometric parameters used for the craniofacial analysis, and measurements made for molar eruption assessment in C57BL/6 mice. (**a**) Experimental protocols D1 and D7: mice were respectively injected from postnatal days 1 and 7. (**b**) Positions on the skull of the different measurements made. (c-f) Methods for measuring molar eruptions in four different situations, corresponding to the control case (**c**), case of alteration to upper first and second molar eruptions (**d**), case of moderate retention of upper and lower molars (**e**) and the case of severe retention of upper and lower molars (**f**). *: mild retention; *: moderate retention; ***: severe retention.

Fourty-day-old wild-type and *Rankl^-/-^* mice were used as positive and negative control groups to analyze the groups of mice that received 4 subcutaneous injections of IK22.5 antibody (25 μg in Phosphate-Buffered Saline for the first and second injections, then 50μg) every 2 days, starting on day 1 (D1 group) and day 7 (D7 group) after birth. The mice were sacrificed 1 month after the last injection (Figure 2a). At least 5 mice per group were assessed without considering the sex parameter based on the fact that all were pre-pubertal.

### 2.3. Micro-CT Analyses

Analyses of bone microarchitecture were performed using a Skyscan 1076 in vivo micro-CT scanner (Skyscan, Kontich, Belgium). Tests were performed after euthanizing the mice in each group. All heads were scanned using the same parameters (pixel size 9 µm, 50 kV, 0.5-mm Al filter, 15 min of scanning). The reconstructions were analyzed using NRecon (v1.7.04) and CTan (v2.0) software (Skyscan, Kontich, Belgium). Three-dimensional visualizations of the heads were obtained using ANT (v1.10.7) and CTvox (v3.0) softwares (Skyscan, Kontich, Belgium).

The morphologies of the skull, and upper and lower jaws were evaluated on the basis of certain vertical, horizontal and sagittal cranial measurements (Figure 2b).

The choice of landmarks was based on a pre-existing analysis [22]. The analysis presented makes it possible to measure several points on the same section in order to facilitate the study.

The molar eruption defects in the mouse models were evaluated according to two parameters: the eruption sequence (normal sequence being: Upper M1, lower M1, upper M2, lower M2, upper M3 and lower M3), and the distance between the occlusal surface of the molars in retention (yellow lines in Figure 2d–f) and the referent occlusal plane in the control mice (red lines in Figure 2c–f).

### 2.4. Histology

The heads were collected from the euthanized mice and fixed in 4% buffered paraformaldehyde (PFA) in phosphate buffered saline 0.1M (PBS) for 48 hours (all chemical products from Sigma-Aldrich, Saint-Quentin Fallavier, France). The heads were decalcified in 4.13% EDTA/0.2% PFA pH 7.4 in PBS for four days in a KOS sw10 (Milestone, Sorisole, Italy). The samples were dehydrated and embedded in paraffin or maintained in a PBS buffer solution at 4 °C before cryostat sectioning. Then, 5-µm-thick frontal sections were stained with Masson’s trichrome and histological images were acquired using a Nano-Zoomer 2.0-RS slide scanner (Hamamatsu Photonics, Hamamatsu, Japan) before being visualized and analyzed with the Nano-Zoomer software.

Tartrate-resistant acid phosphatase staining was performed as previously described [14] to identify the multinucleated osteoclast cells (all chemical products from Sigma-Aldrich, Saint-Quentin Fallavier, France).

### 2.5. Statistics

Statistical analyses were carried out using Prism (6.01) software (GraphPad Software, La Jolla, CA, USA). The Fisher test was used to analyze the data relative to patient distribution in the two groups of molar retention. The Student’s *t*-test was used for Tweed’s data analyses and the Fisher test for Delaire’s data analyses. The one-way ANOVA test followed by the Tukey post-hoc test were used to analyze the mouse craniofacial morphometric data.

### 2.6. Study Approvals

The human study was carried out in accordance with a declaration made to the French “Commission National de l’Informatique et des Libertés” (French data protection authority, number g8w2314849D).

The mouse study was validated by the French ethical committee of the “Pays de la Loire” (CEEA-PdL-06) and authorized by the French ministry of agriculture and fisheries (authorization numbers 11208-2017083115577055 and 18415-201901101823350-v2).

## 3. Results

### 3.1. Craniofacial Morphology Features of Patients with Primary Retention of Molars

In order to analyze the potential relationship between Primary Retention of Molars (PRM) and craniofacial growth alterations, the craniofacial phenotypes of patients at the Orthopedics-Dentofacial Department of the hospital “Pitié-Salpêtrière” (AP-HP, Paris, France) presenting with PRM (24 patients) were compared with those of patients from the same Department with impactions or mechanical retentions (18 patients constituting the control group C). The two groups of patients were scrutinized for significant recruitment differences (Table 1). No difference was observed concerning the sex ratio, ethnic group distribution, uni- versus bi-laterality of the retention, position in the osseous (Supra, Juxta or Infra) or the presence of associated dental anomalies. Significant differences were nevertheless observed regarding the family background (*p* = 0.014), involvement of solely one or both dental arches (*p* = 0.033), teeth involved (*p* = 0.0008), radiographic visibility of the eruption pathway (*p* = 0.0087) and classification of the malocclusion angle (*p* = 0.0043).

**Figure 3:**
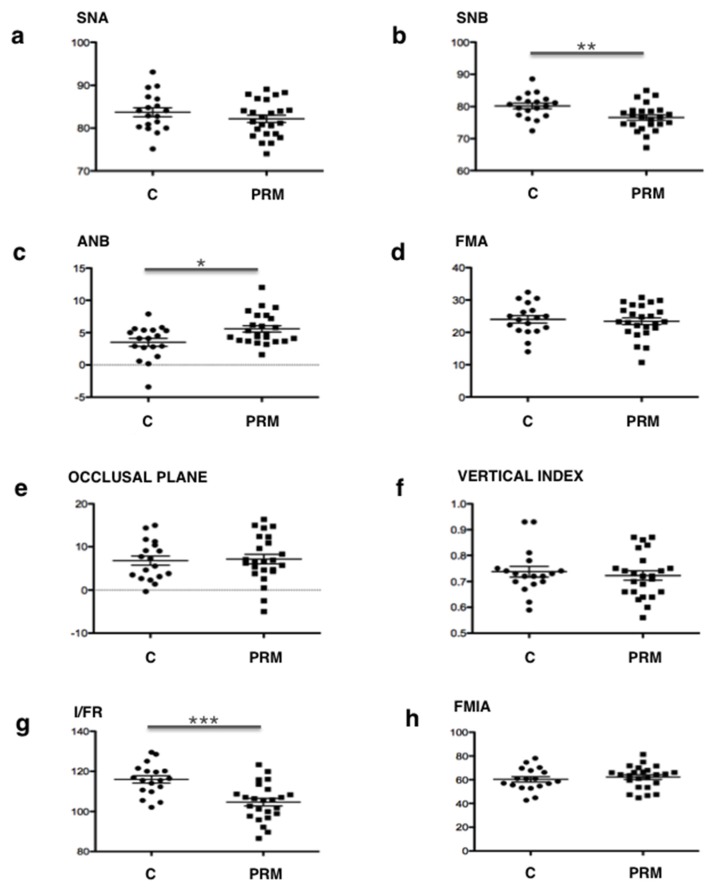
Comparative analysis of Tweed’s craniofacial measurements between control patients “C”, and patients with primary retention of molars «PRM». Relative position maxillary-mandible: (**a**) SNA; (**b**) SNB and (**c**) ANB. Vertical analysis: (**d**) FMA; (**e**) occlusal plane and (**f**) vertical index. Dental analysis: (**g**) I/FR (upper incisor) and (**h**) FMIA (lower incisor). All statistically significant differences evidenced by the Student’s *t*-test are presented. *: *p* < 0.05; **: *p* < 0.01; ***: *p* < 0.001.

Tele-X-ray studies of these patients were conducted using both Tweed’s and Delaire’s cephalometric analyses. The Tweed’s analysis (Figure 3 and Table 2) evidenced statistically significant differences between the two groups of patients concerning the SNB angle (*p* = 0.006) with an average of 76.6° for the PRM group versus 80.2° for the C group (norm = 80°), the ANB angle (*p* = 0.0112) with an average of 5.6° for the PRM group versus 3.5° for the C group (norm between 0° and 4°), and the I/FR angle (*p* = 0.0001), with an average of 104.7° for the PRM group versus 116° for the C group (norm = 107°). No statistically significant difference was found between the two groups for any other measurements (Figure 3 and Table 2).

For the Delaire’s analysis, in the PRM group, most patients presented with skeletal class II (96%), the others (4%) with class III, whereas no patient was of skeletal class I (Table 3). In the C group, 67% of patients were in class II, 22% in class I, and 11% in class III. The distribution of skeletal classes between the two groups was found to be statistically different (*p* = 0.0378). With regard to mandible position, in the C group, 39% of patients presented with retrognathism, whereas this figure was 75% for patients with primary retentions (*p* = 0.0274). Interestingly, of the skeletal class II patients in the C group, only 36% presented with retrognathism, whereas in the PRM group, 78% of the patients in skeletal class II had an associated retrognathism (Table 3).

For the morphology of the mandible, in the patients in the C group, the gonial angle was closed at 22% versus 17% for the patients in the PRM group (*p* = 0.697). In the PRM group, the mandibular dimensions were smaller than for the C group, and statistically significantly different for the ramus (*p* = 0.0266) and mandible length (*p* = 0.041). In fact, there were 67% of cases of short ascending ramus, 39% of short mandible body and 79% of brachygnathism found in patients with primary retention of molars, as opposed to, respectively, 39%, 33% and 50% in the subjects in the control group (Table 3).

Regarding the lower height, no statistically significant difference was found between the two groups (*p* = 0.603). Nevertheless, subjects with primary retention of the molars still showed a slightly increased height in the lower stage in 58% of cases, compared to 44% for the subjects in the control group.

Regarding the dental measurements, 83% of patients with primary retentions had palatoversion of the upper incisors versus 44% for the patients in the control group (*p* = 0.018). However, for the lower incisors, no significant difference was found between the two groups.

### 3.2. Impact of Transient Inhibition of RANKL on Molar Eruption and Craniofacial Morphometric Parameters in Mice

In order to analyze the potential implication of transient inhibition of RANKL signaling in the occurrence of PRM, two mouse models were used, consisting of a series of four injections every two days of a powerful RANKL blocking antibody (IK22.5) over two distinct periods starting on postnatal day 1 (D1 group) or postnatal day 7 (D7 group). The impact of these transient inhibitions of RANKL signaling on the eruption of the molars and craniofacial growth was analyzed one month after the last injection using microCT and histology with wild-type and *Rankl^-/-^* mice as the positive and negative controls groups, respectively.

**Figure 4:**
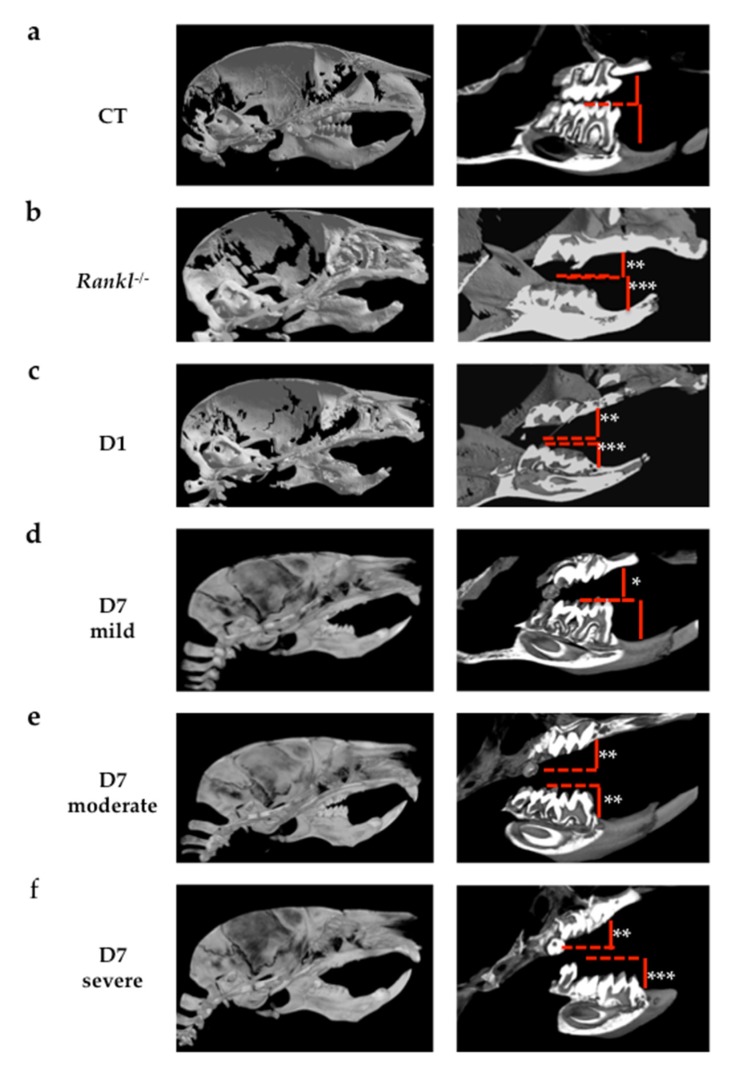
Analyses using micro-CT scans of molar eruption disturbances on different groups of C57BL/6 mice. Images of a representative mouse from each group are given. (**a**) Wild-type mouse group serving as the positive control for molar eruption. (**b**) *Rankl^-/-^* mouse group serving as the negative control for molar eruption. (**c**) Mouse injected with IK22.5 from day 1 (D1 group); (**d**) mouse injected with IK22.5 from day 7 with mild eruption retention (D7 mild group); (**e**) mouse injected with IK22.5 from day 7 with moderate eruption retention (D7 moderate group); (**f**) mouse injected with IK22.5 from day 7 with severe eruption retention (D7 severe group). Red lines correspond to the referent occlusal plane observed in the control mice. *: mild retention; *: moderate retention; ***: severe retention.

For the impacts of transient inhibitions of RANKL on molar eruption in mice, the eruption profiles obtained with micro-CT in the different groups of mice are presented in Figure 4.

Interestingly, while the mice in the D1 group evidenced a homogenous phenotype (Figure 4c), those from the D7 group presented with variable eruption patterns, with several levels of defect concerning the first and second molars that were classified as mild, moderate and severe (Figure 4d–f). In the mice in the D1 group, the presence of severe primary retentions of all the first (M1) and second (M2) molars that were in an intraosseous position was observed. The M1 appeared in all cases more severely affected than the M2. Despite the severity of the M1 and M2 retentions, the third molars (M3) were clearly unaffected (Figure 4c). Remarkably, in all the mice in the D7 protocol, the M3 were also not affected, which validates the fact that the two chosen periods of RANKL inhibition target were only M1 and M2. In the D7 mice with a mild retention pattern, the upper first (M1) and second (M2) molars were retained while the lower molars did not appear to be affected (Figure 4d). In the D7 moderate group (Figure 4e), all the M1 and M2 showed eruption alterations, although with different levels of severity. The lower molars showed an eruption delay or a secondary retention, with infraocclusion as the molars were visible in the oral cavity, while the upper molars had primary retention and remained intraosseous. Upper second molars were clearly the most severely affected. In the D7 severe group (Figure 4f), all M1 and M2 were retained, almost all intraosseous except for the cuspid points of the lower molars. Again, the upper molars were more severely affected than the lower ones.

**Figure 5:**
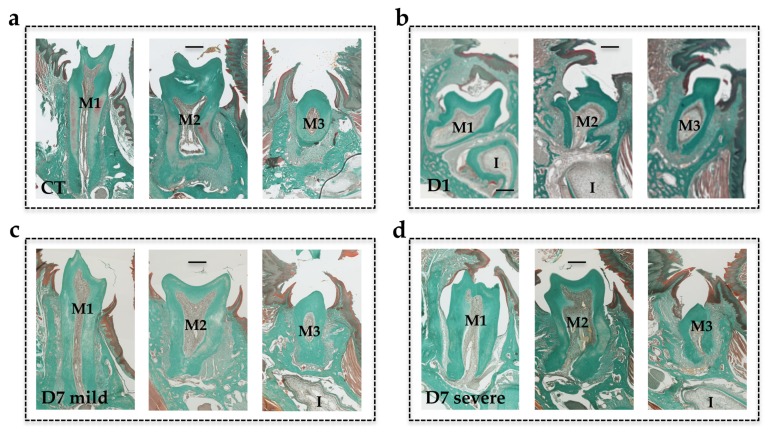
Masson’s trichrome staining of frontal sections of mouse heads in the planes of the first, second and third lower molars. (**a**) Wild-type mouse group (Control CT); (**b**) mouse injected with IK22.5 from day 1 (D1) group; (**c**) mouse injected with IK22.5 from day 7 with mild eruption retention (D7 mild) group; (**d**) mouse injected with IK22.5 from day 7 with severe eruption retention (D7 severe) group. M1: first lower molar; M2: second lower molar; M3: third lower molar. Scale: 200 µm.

For more in-depth study of the effects of the transient inhibition of RANKL on the mandible alveolar bone remodeling necessary for the eruption of molars, and on mandible molar organogenesis, a histological analysis was performed. Masson’s trichrome staining (Figure 5) revealed that compared to mice from the wild-type group, those from the D1 group had the most severe phenotype, with the first and second molars in retention and severe tissue disorganization. As shown by micro-CT, the third molars appeared histologically unaffected regardless of the protocol considered. The mice from the D7 protocol showed variable and heterogeneous phenotypes with mild, moderate and severe forms (Figure 5c–d). In severe forms, the molars retained were mainly the first molars, but tissue disorganization was observed for both the first and second molars.

**Figure 6:**
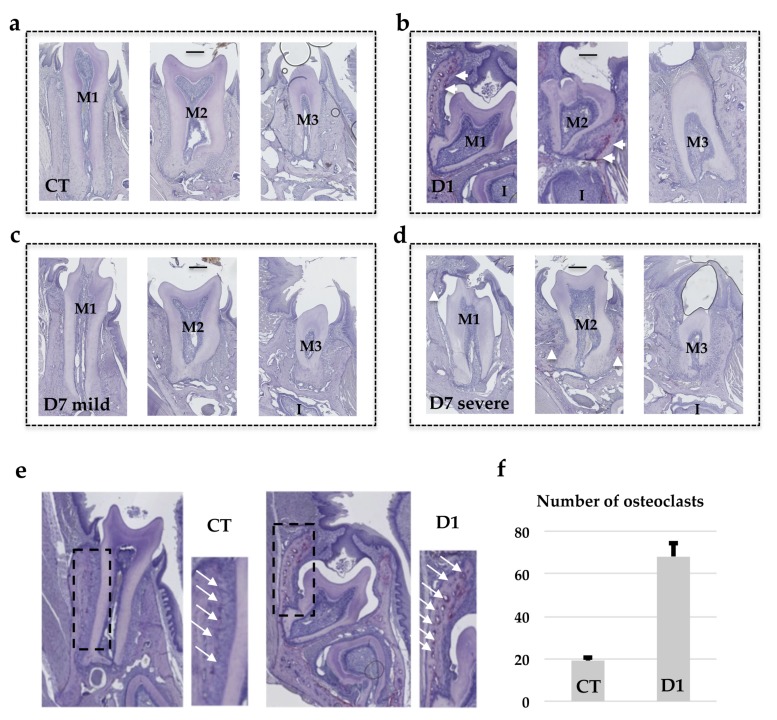
TRAP staining of frontal sections of mouse heads in the planes of the first, second and third lower molars. Comparative quantitative analysis of the number of TRAP positive cells (white arrowheads) at the surface of the vestibular alveolar bone between mice in the wild-type group and those injected with IK22.5 from day 1 (D1) group. (**a**) Wild-type mouse group (Control CT); (**b**) mouse injected with IK22.5 from day 1 (D1) group; (**c**) mouse injected with IK22.5 from day 7 with mild eruption retention (D7 light) group; (**d**) mouse injected with IK22.5 from day 7 with severe eruption retention (D7 severe) group. M1: first lower molar; M2: second lower molar; M3: third lower molar. Scale: 200µm. (**e**) Representative positions (rectangles) of alveolar bone areas used after enlargement to count the TRAP positive cells in the control and D1 groups. The white arrows show the TRAP positive cells present at the surface of the alveolar bone adjacent to the periodontal ligament. (**f**) Graphic representation of the quantification made in the areas presented in (e) for all mice from the CT and D1 groups. The bar represents the average with the standard deviation calculated for each group of mice.

Remarkably, TRAP histo-enzymology staining (Figure 6) revealed that the number of TRAP positive cells was higher in the alveolar bone when the molar phenotype was less severe. The higher TRAP staining was more visible around the first and second molars of the mice in the D1 group (Figure 6b) than around those of the mice in the D7 group with the severe phenotype. TRAP histo-enzymology in the coronary region also made it possible to observe the formation of an eruption pathway in the mice in the D1 group, despite the intra-osseous retention of the molars (Figure 6e).

In order to assess the effects of transient inhibitions of RANKL on craniofacial development in mice, the morphologies of the skull and upper and lower jaws were characterized using the measurements of certain horizontal, vertical and sagittal morphometric parameters. Similar measurements were obtained for wild-type and *Rankl^-/-^* mice to serve respectively as the negative and positive controls with regard to RANKL invalidation. 

**Figure 7:**
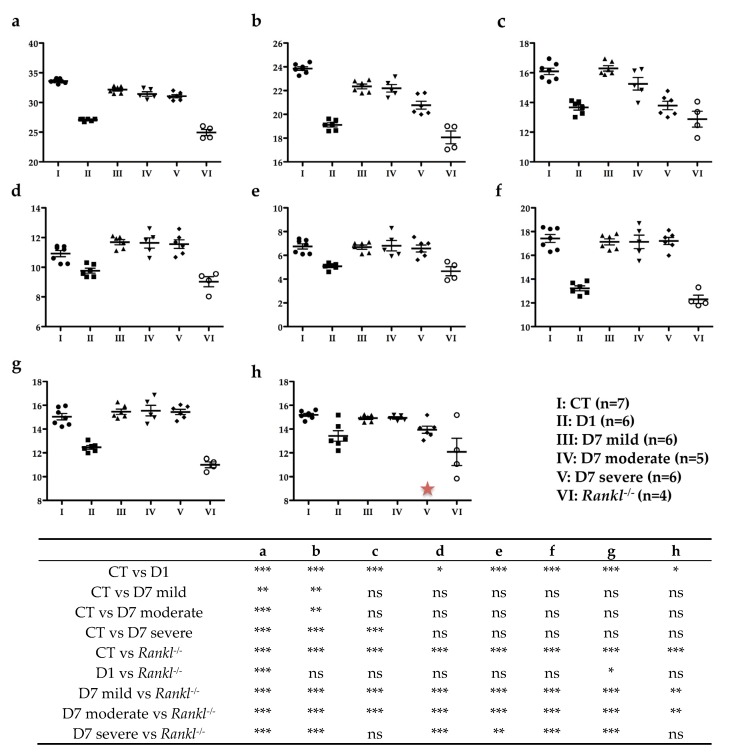
Comparative analysis of the different cranial measurements made for the six groups of mice. (**a**) Total skull length; (**b**) cranial vault length; (**c**) facial region length; **(d**) middle cranial length; (**e**) facial height; (**f**) inter-zygomatic arch width; **(g**) upper mandible length; (**h**) lower mandible. All statistically significant differences between the groups, evidenced by an ANOVA test with a Tukey post-hoc test, are presented in the table. *: *p* < 0.05; **: *p* < 0.01; ***: *p* < 0.001. The red star in (h) indicates a tendency to decrease compared to the control group.

When compared to the reference wild-type group, all the measurements revealed a statistically significant difference (*p* ≤ 0.001) in the positive control *Rankl^-/-^* group (Figure 7).

Concerning the D1 group, all mice showed a reduction in the craniofacial measurements compared to the mice in the wild-type group, which appeared similar to the reduction observed in the mice in the *Rankl^-/-^* group (Figure 7). A significant decrease was reported for the same measurements as for the *Rankl^-/-^* group. Surprisingly, a significant difference was observed between the D1 and *Rankl^-/-^* groups for total skull length (a; *p* < 0.001) and upper mandible length (g; *p* < 0.05).

Regarding the mice in the D7 protocol, the measurements were made taking into consideration the different phenotypes in terms of severity. Interestingly, only two parameters were affected in all the mice in the D7 group compared to the mice in the wild-type group: total skull length (a) and cranial vault length (b). Facial region length (c) was also significantly affected compared to the mice in the wild-type group, but only for the mice in the D7 severe group (Figure 7).

Finally, it seems that the parameters in the mice most phenotypically affected by transient RANKL inhibitions tended toward the values observed for the *Rankl^-/-^* group, whereas the parameters remained similar to the wild-type group’s values in less affected mice. 

## 4. Discussion

The primary retention of molars (PRM) observed in clinic corresponds to a still unexplained absence of molar eruption, despite a large eruption pathway and no physical obstacle [2]. Interestingly, this is similar to the situation previously reported [15] as being associated with transient inhibition of RANKL signaling during the first postnatal week, raising the question of the involvement of RANKL signaling in the occurrence of primary retention of molars. The present study was conducted in order to validate the hypothesis that transitory perturbations to RANKL signaling during growth are involved in the occurrence of PRM, and that these perturbations are correlated with a craniofacial skeleton growth pattern. In order to demonstrate such involvement, the experimental strategy used was based on a double methodology; a clinical study consisting of the characterization of the craniofacial phenotypes of patients with PRM, and an experimental study in mice consisting of transiently blocking RANKL signaling and analyzing the consequences on molar eruption and craniofacial growth, researching for similar features.

Regarding the association in human patients of primary retention of molars with a wider spectrum of craniofacial skeleton growth alterations, two cephalometric comparative analyses of patients with primary retention of molars and patients with mechanical impactions were carried out. The control group corresponding to patients with mechanical molar impactions was chosen in order to distinguish the craniofacial morphometric features associated with the primary retention of molars from those simply linked to molar retention. A total of 42 patients with molar retention were included in our analysis, 24 with primary retention of molars (including 15 with a PFE) and 18 with mechanical retention. A third group of 18 patients from the same department without retention was also studied to confirm that the global population follows the norm of the Tweed’s analyses (Table 4). Tweed’s analysis used standard measurements to make it possible to compare the patient with a cephalometric standard: the quantitative study was thus easier. Delaire’s analysis made it possible to qualitatively highlight the craniofacial typology of the patient and to confront it with what could have been its structural or dento-skeletal optimum. Interestingly, the use of two different analyses made it possible to compare the results obtained and to assess their coherence.

The results of the qualitative study (Delaire’s analysis) showed a statistically significant difference in the distribution of skeletal classes and the antero-posterior position of the mandible between the two groups. A higher prevalence of skeletal classes II (96%) associated with retromandibulia in 78% of cases was found in the group of primary retentions compared to the control group. These results are consistent with those found in Tweed’s analysis. An ANB angle of more than 4° represents, according to Tweed, skeletal class II. The average ANB angle in the group of primary retentions (5.58°) was not only higher than this norm, but was also significantly different from that of the control group (3.5°). Similarly, patients with primary retentions had retromandibulism (mean SNB = 76.6°, which is lower than the 80° norm) compared to patients from the control group (*p* = 0.006).

Few articles in the literature have reported peculiar craniofacial skeleton patterns in patients with eruption defects. In 2010, Frazier Bowers et al. [23] counted two skeletal class III cases out of four patients with primary failure of eruption (PFE) and in 2013, Rhoads et al. [24] reported 18 skeletal class III cases in the 58 patients included in the study, and found a more frequent tendency for class III when the patient had PFE. However, analyses have focused on patients with PFE and not on patients with other primary retention alterations, and these patients were divided into two groups: 11 subjects with a genetically-confirmed PFE (*PTHR1* mutations) presenting with skeletal class III in 63.7% of the cases, and 47 subjects with clinically-diagnosed PFE that presented with skeletal class III in only in 23.4% of the cases. Surprisingly, other skeletal classes were not listed. It should also be noted that no specific cephalometric analyses were reported in this study: the results were determined from "good quality" clinical images by reporting the Dental Angle class, and profile tele-X-ray when it was available in the orthodontic file, without specifying the method of analysis [24]. Finally, in 2016, Sharma et al. [25] found seven skeletal class III cases in the 15 patients studied with PFE. This study relied on orthodontic records but without specifying the protocol or the documents used. A significant divergence was thus noted between the results of our study and those in the literature. This divergence can be explained by the fact that the populations studied are partly different. Only patients with PFE were analyzed in the literature whereas in the present study all patients with primary retention of eruption (including PFE (n = 15) and other alterations (n = 9)) were included. Moreover, none of the studies published have reported the use of cephalometric analysis from lateral tele-X-ray, whereas in our study, two different analyses (Delaire and Tweed) were used to determine the skeletal pattern.

To return to the results of our study, for the vertical craniofacial measurements, neither of the two cephalometric analyses used revealed any significant difference between the two groups. However, regarding the dental measurements, both Delaire’s and Tweed’s analyses found statistically significant palatoversion of the upper incisor in patients with primary retentions (mean I/FR = 104.7°) compared to the control patients. These observations are in agreement with those obtained clinically (in the description of the population). In fact, patients in the "primary retention" group had a higher prevalence of class II.2 associated with anterior overbite (75%) compared to the control group. Although this is not a skeletal measurement, it is interesting to note that this malocclusion was particularly preponderant in these patients.

These results led us to assume the existence of a particular craniofacial phenotype in patients with primary retention of molars: a retromandibular skeletal class II typology associated with reduced mandibular dimensions (short ascending ramus, short mandible body and brachygnathism), which manifests itself at the dental level in a class II/2 with palatoversion of the upper incisors and anterior overbite. This association reinforces the link between craniofacial growth and dental eruption and will be the starting point for further research.

Concerning the study in mice on the consequences of the transient invalidation of RANKL signaling on molar eruption and craniofacial growth, two periods of RANKL inhibition were chosen, from postnatal days 1 to 9 (D1 group) and 7 to 15 (D7 group). The aim was to mainly target first and second molar eruptions respectively [13]. This type of mouse model was chosen for its transitory aspect, making it possible to clearly evidence the importance of the timing of RANKL inhibition in the occurrence of primary retention of molars. Regarding the validity of such a model for human studies, this model is a systemic transitory invalidation of RANKL with molars in the four quadrants affected, corresponding to a minority of the cases in human patients (the most severe). Regardless, the clearly established link between RANKL transitory invalidation during growth and the primary retention of molars in a wider context of craniofacial morphometric alterations strongly supports the fact that local transitory invalidation of RANKL may be at the origin of localized primary retention of molars in human.

Regarding molar eruption, significant heterogeneity was observed in the alterations to the first and second molars in the mice in the D7 group, while for all mice in the D1 group, severe first and second molar retentions were reported. Interestingly, the severity of the phenotype was less important and less generalized in the mice in the D7 group than in those in the D1 group. These observations suggest the presence of a temporal window for RANKL signaling that is of major importance for both first and second molar eruptions, and that encompasses most of the first postnatal week and part of the second week. The fact that the first and second molars were respectively the most affected teeth in the D1 and D7 groups nevertheless confirms that each tooth has its own temporal window. Interestingly, regardless of the inhibition protocol considered, the third molars always erupted, revealing a significant delay in the third molars eruption window compared to the overlapping first and second molar windows.

Remarkably, TRAP histo-enzymology staining revealed that one month after the end of treatment, the number of TRAP positive cells was higher in the alveolar bone when the molar phenotype was more severe. The higher TRAP staining was visible around the first and second molars in the mice in the D1 group then around those in the mice in the D7 group with the severe phenotype. The TRAP histo-enzymology also made it possible to observe, in the coronary region, the formation of an eruption pathway in the mice in the D1 group despite the intra-osseous retention of the molars. These histological results demonstrated that transient invalidation of the RANKL signaling had an impact on the molar eruption and root formation processes in which the intensity (for each molar) was dependent on the period during which this inhibition was effective, and was independent of the later formation of an eruption pathway. Interestingly, decreasing the period (window) of inhibition in the D1 protocol by reducing the number of injections of the RANKL blocking antibody from 4 to 3 and 2 has previously been shown to gradually reduce the dento-alveolar phenotype [15], underlining the fact that the length of the inhibition period was also an important criterion to be taken into consideration, in addition to the inhibition initiation time.

Regarding the morphometric craniofacial parameters, the decrease in craniofacial measurements was related to the level of alteration in the bone remodeling. Constitutive inhibition of RANKL (*Rankl^-/-^*) leads to osteopetrosis, with the presence of significant growth retardation, alterations to bone metabolism due to a decrease in osteoclastic differentiation, perturbations to dental and bone cell communications, and alterations to dental eruption [21,26]. Interestingly, transient inhibition of RANKL during the first postnatal week induced craniofacial morphometric defects very similar to those observed in *Rankl^-/-^* mice, whereas inhibition during the second postnatal week induced a less severe phenotype with most of the parameters measured similar to those of the wild-type mice. The two craniofacial morphometric parameters that were the most affected in relation to defective molar eruption were total skull length and craniofacial vault length. In the case of RANKL signaling perturbations during growth, when tooth retention or tooth eruption delay was observed, a reduction in these craniofacial morphometric parameters was effectively suggesting, as hypothesized, that primary molar retention was part of a more global craniofacial growth alteration associated with transitory disruption in RANKL signaling.

In this context, the intervention of environmental factors that perturb RANKL signaling during growth may have consequences on dental and craniofacial development, with heterogeneity that depends on the stage of intervention of these factors and their local or systemic application. In the case of a systemic intervention, craniofacial growth and dental eruption were thus closely related processes that depended on osteoclastogenesis, itself genetically regulated in time and space by RANKL signaling [11,27,28]. Interestingly, the transient inhibition of RANKL could also alter other signaling pathways involved in alveolar bone modeling and root formation, such as the tumor growth factors/bone morphogenetic protein (Tgfβ/Bmp) pathway, Wingless/β-catenin (Wnt/β-catenin) pathway, fibroblast growth factor (Fgf) pathway, Sonic hedgehog (Shh) pathway or insulin-like growth factor (IGF) pathway [29,30], leading to an alteration to root formation and an absence of eruption despite the presence of osteoclasts.

Apart from environmental factors, the expression of intrinsic/genetic factors/function alterations may also be the source of RANKL signaling perturbations, leading to primary retention of molars and craniofacial development defects, as already shown for parathyroid hormone-related peptide (PTHrP). The PTHrP signaling pathway has been strongly implicated in the retention of molars [8,31]. Functionally, PTH1R is strongly expressed by osteoblasts adjacent to the dental germ, whereas PTHrP is expressed by the epithelial cells in the dental lamina and the stellate reticulum just before the formation of the eruption pathway [32,33]. PTHrP/PTH1R signaling is therefore implicated in the formation and activation of the osteoclasts necessary for tooth eruption through stimulation of RANKL expression by the osteoblasts.

Globally, all factors that can perturb RANKL signaling during growth, regardless of their origins, may cause primary retention of molars as part of a wider spectrum of craniofacial defects, mainly characterized by reduction in growth in the horizontal dimension, coherent with the II.2 skeletal class observed in patients diagnosed with primary retention of molars.

## 5. Conclusions

When combining all the results obtained from patients and from our mouse models of RANKL signaling invalidations, we can conclude that primary retention of molars is part of a broader craniofacial skeleton phenotype, the origin of which may lie in transitory alteration to RANKL functions during the initial stage of dental root elongation and tooth eruption. Interestingly, this phenotype may eventually become a predictive clinical sign for the primary retention of molars. Regardless, further studies will be needed to integrate the notion of systemic versus local in the link between RANKL invalidation, primary retention of molars and craniofacial morphometric alterations.

## Figures and Tables

**Table 1:** Analysis of patient distribution in terms of age, sex, ethnicity, family history, uni- or bilateral characteristics, type of arcade affected, type of tooth affected, supra- or infra-crestal position, presence of a pathway of eruption (for infra-crestal molars), other associated anomalies, and the dental diagnosis. *, ** and ***: Significant variation evidenced using the Fisher test respectively *p* < 0.05, *p* < 0.01 and *p* < 0.001.

		C Group N = 18	PRM Group N = 24	*p* (Fisher Test)
**Sex**	F	9	10	0.756
	M	9	14	
Ethnic group	Caucasian	7	15	0.063
	African	6	5	
	North-African	2	4	
	Asian	3	0	
Family history	Presence	0	7	0.014*
	Absence	18	17	
Uni/bilateral	Unilateral	9	9	0.532
	Bilateral	9	15	
Dental arches	1 arch	13	9	0.033*
	2 arches	5	15	
Type of teeth	1st Molar	0	2	0.0008***
	2nd Molar	18	13	
	1st + 2nd Molars	0	9	
Supra/infra -crestal	Supra.	9	11	1
	Juxta.	2	4	
	Infra.	7	9	
Eruption pathway		*On 7 infra*	*On 9 infra*	0.0087**
	Visible	1	8	
	Not visible	6	1	
Associated alterations	Total	4	8	0.506
	Impactions	4	4	
	Inclusions	0	1	
	Agenesis	0	2	
	Ankylosis	0	1	
	No	14	16	
Dental diagnosis	Class II.2	5	18	0.0043**
	Other	13	6	

**Table 2:** Tweed’s cephalometric analysis. Comparison between patients in the Control group C and the Primary Retention of Molars group PRM. *, ** and ***: Significant variation evidenced by Student’s *t*-test respectively *p* < 0.05, *p* < 0.01 and *p* < 0.001.

		Tweed’s Norm	Group C	Group PRM				
			Mean ± Standard Deviation		Mean Difference	CI 95%	t	*p* (Student’s *t*-Test)
Sagittal skeletal measurements	SNA	82 ± 2	83.73 ± 1.0460	82.21 ± 0.8616	1.515 ± 1.345	[−1.203; 4.234]	1.126	0.2667
	SNB	80 ± 2	80.22 ± 0.8810	76.61 ± 0.8511	3.610 ± 1.245	[1.094; 6.125]	2.900	0.0060**
	ANB	2	3.506 ± 0.6186	5.583 ± 0.4932	−2.078 ± 0.7816	[−3.657; −0.4982]	2.658	0.0112*
Vertical skeletal measurements	FMA	25 ± 3	24.02 ± 1.145	23.46 ± 1.033	0.5597 ± 1.552	[−2.576; 3.696]	0.3607	0,7202
	Occlusal plane	10.14	6.800 ± 1.068	7.138 ± 1.109	−0.3375 ± 1.581	[−3.532; 2.857]	0.2135	0.8320
	Vertical index	0.65–0.75	0.7372 ± 0.02042	0.7229 ± 0.01756	0.01431 ± 0.0269	[−0.0401; 0.068]	0.5317	0.5979
Dental measurements	FMIA	67 ± 3	60.27 ± 2.239	62.17 ± 1.922	−1.900 ± 2.947	[−7.855; 4.055]	0.6448	0.5227
	IMPA	90 ± 3	95.73 ± 2.379	94.37 ± 1.616	1.361 ± 2.777	[−4.252; 6.974]	0.4901	0.6268
	I/FR	107	116.0 ± 1.826	104.7 ± 1.890	11.33 ± 2.697	[5.883; 16.79]	4.202	0.0001***

**Table 3:** Delaire’s cephalometric analysis. Comparison between patients in the Control group C and the Primary Retention of Molars group PRM. *: Significant variation evidenced by Fisher test *p* < 0.05.

			Group C	Group PRM	*p* (Fisher Test)
Sagittal	Skeletal class	Class I Class II Class III	4 12 2	0 23 1	0.0378*
	Mandible position	M. retrusion M. protrusion	7 11	18 6	0.0274*
Mandible morphology	Ramus	Dolicho-ramus Brachy-ramus Normal	7 7 4	7 17 0	0.0266*
	Corpus (mandible body)	Dolico-corpus Brachy-corpus Normal	12 6 0	11 11 2	0.390
	Gonial angle	Opening Closing Normal	13 4 1	19 5 0	0.697
	Mandible length	Dolico-mandible Brachy-mandible	9 9	4 20	0.041*
Vertical	Lower height	Increase Decrease Normal	8 5 5	14 6 4	0.603
Incisors	Upper incisor	Palatoversion vestibuloversion	8 10	20 4	0.018*
	Lower incisor	Linguoversion Vestibuloversion	7 11	11 13	0.757

**Table 4:** Tweed’s cephalometric analysis of a group of patients without molar retention from the same orthodontic department as the C and PRM groups. Occlusal plane and I/FR (underlined) are the only measurements distinct from the norm, which can be explained by dental maxillary disharmony in these patients.

		Tweed’s Norm	Group without Retention (n = 18)
			Mean ± Standard Deviation
Sagittal skeletal measurements	SNA	82 ± 2	82.41 ± 3.09
	SNB	80 ± 2	80.66 ± 2.81
	ANB	2	2.01 ± 1.14
Vertical skeletal measurements	FMA	25 ± 3	24.44 ± 3.53
	Occlusal plane	10.14	5.27 ± 3.09
	Vertical index	0.65–0.75	0.69 ± 0.065
Dental measurements	FMIA	67 ± 3	61.07 ± 4.54
	IMPA	90 ± 3	94.47 ± 4.36
	I/FR	107	115.82 ± 5.64
